# Reframing a debate in chiropractic

**DOI:** 10.1186/s12998-021-00401-5

**Published:** 2021-11-03

**Authors:** Henry Pollard

**Affiliations:** Department of Chiropractic, School of Medical and Applied Sciences, CQUniversity, Brisbane, Australia

**Keywords:** Chiropractic, Training, Competency, Education, Undergraduate, Post graduate, Student, Specialist, Generalist, Title, Licensure, Wellness, Lifestyle medicine, EBM

## Abstract

The chiropractic profession is 125 years old and has evolved a culture beset with internal conflict. The internal ructions have been particularly noticeable during the last 20 years. The recent resignation of the entire World Federation of Chiropractic Research Committee has again focussed the conflicting views and goals of the “wellness” and “evidence” factions within the profession. These polarising viewpoints are worsening to the degree that there are calls for the profession to break into two separate entities. Key to the recognition of the differences within the profession is the recognition of title for particular sub populations of patients presenting to chiropractors. For many of the sub populations such as sport or paediatrics there has grown appropriate post professional specialist educational training sometimes leading to a protected title. However, this is not occurring in that group of practitioners that choose to focus on wellness care. A recommendation is made that wellness chiropractic be viewed as a post professional specialty program within chiropractic, as it is in medicine and elsewhere, and that recognition follow after appropriate post professional educational programs have been completed, as is customary in the other special interest groups. In order to do so, consensus will be required from all stakeholders within the profession on the level, scope and depth of such programs. Furthermore, it is possible that different jurisdictions around the world may require different post graduate educational levels based on local competitive, legal and professional circumstances. In such cases, transitioning to the higher level over a period of time may be undertaken. Recognition of the wellness specialty by the profession would allow for vertical integration with other healthcare providers as well as help bridge a gap between the entrepreneur and academic groups that would be responsible for creating these programs at tertiary education institutions. Finally, should these programs acquire evidence to underpin them, a process that would be taught within the programs, it is likely that recognition of an extended scope of practice would occur increasing the appeal of chiropractic to the public.

## Background

The wellness traditional model of chiropractic care and those who represent it are a part of the total scope of care provided by chiropractors. The division between that group and that represented by the “evidence-based/science” end of the profession continues to disrupt the direction, inclusiveness and development of the whole profession. This divide has expanded to the degree that there are open calls for the profession to split into two groups [1]. Despite these internal professional ructions, it is likely that the chiropractic profession should focus less on identity driven issues and more on patient centred issues such as the consumer expectation and the healthcare environment of a modern healthcare profession.

This paper will discuss antecedent factors associated with the opposing viewpoints and propose a solution to the impasse based on solutions in professional structure and education that already exist in other healthcare professions. In doing so, it will also discuss the training of graduate and post graduate generalist and specialist training programs in chiropractic.

### Wellbeing and lifestyle medicine

The term “wellness” is frequently used within the chiropractic profession [2, 3] and is defined as: a patient-centred process, which emphasises the development of health promotion and disease prevention, pursuit of wellbeing with a focus on the spine [4, 5]. Wellness approaches vary from early Palmerian approaches to more modern concepts typified at colleges and universities worldwide including Palmer [6, 7]. Wellness has also come to represent the “maintenance care” provided by many chiropractors whilst acquiring these goals [8, 9], which focuses on a spine-based focus on illness prevention and health promotion, identity and general wellness (wellbeing) of patients [10]. A cornerstone idea of which is to prevent a myriad of health diseases and complaints whilst pursing this spine focussed goal of wellbeing or wellness [11].

The practice of wellness care, however, is not unique to chiropractic [12]. Medicine, for example, often refers to “wellness” as “wellbeing” in order to reflect the broader psychological component of health [13].

It seems that many of the goals encapsulated in “chiropractic wellness” are present in the medical and allied health arenas of the sub-discipline referred to as Lifestyle Medicine (LM) [14] albeit without a spine focus. “Lifestyle Medicine is an evidence based interdisciplinary, whole-system approach to the prevention and reversal of chronic and lifestyle-related diseases through the modification of the behavioural, social and environmental drivers. It involves clinicians, public health professionals, healthcare executives, researchers, scientists and educators working together to prevent, manage and treat conditions that result from:

- Physical inactivity
- Poor diet or nutrition
- Smoking
- Alcohol overconsumption
- Chronic stress
- Anxiety
- Poor or inadequate sleep
- Social isolation
- Loss of culture and identity
- Other influences of society and environment” [15].

To this definition of LM many chiropractors would add a focus on musculoskeletal (MSK) health and others would additionally add a focus on spinal hygiene [16]. It is this spinal and neuromusculoskeletal focus that presents a unique potential contribution by chiropractors to the LM discipline if that focus could be supported by evidence.

Spinal hygiene is a concept derived from dental hygiene practice [16] that encapsulates the provision of regular preventative chiropractic care that aims to prevent the development of spinal complaints and improve health. Often, it is adopted by entrepreneurial groups using extended care regimens, including “maintenance care” [17]. Whilst the over servicing aspect of maintenance care has been debated [18], recent evidence suggests a role for maintenance care in some sub-populations of patients presenting for chiropractic services [19, 20]. Yet, this modern evidence is for maintenance care provided without the wellness focus [21]. However, entrepreneurial activity also presents a key focus within the maintenance care approach; specifically, that spinal hygiene presents myriad opportunities to sell products and partner with like-minded companies [17]. This concept appears somewhat inconsistent with the self-help approach to the management of chronic disease encapsulated within LM [14].

Problems with the entrepreneurial activities of some chiropractors with a wellness focus have been identified [22–25]. In a spectrum of basic required business skills identified as lacking in most chiropractic educational programs [26] through to unethical predatory practices [27, 28] there should be a clear delineation between required business skills and unethical behaviour [29]. The provision of such training in pre professional programs may go some way to address shortcomings in the business ethics whilst providing acceptable and ethical practices for the profession [30].

While in its infancy, the pursuit of wellness objectives has recently gained much attention within the medical profession; for example, a formal lifestyle medicine training program was launched in 2006 by Loma Linda University, which was subsequently followed by the development of the first peer-reviewed professional journal dedicated to Lifestyle Medicine, the American Journal of Lifestyle Medicine, in 2007 [31]. It is the opinion of this writer that spine focussed MSK wellness care presents an opportunity for chiropractic to evolve and take leadership in this new and emerging sub-discipline. However, the entrepreneurial goals need to be separated from the health goals in order for this concept to progress.

### Wellness versus evidence: a false premise

Years of stagnation in the management of specialist chiropractic practitioners and their post professional education has contributed to the development of a strong schism within an increasingly binary profession, locked within a downward spiral. Subsequently, two vocal groups focussing on either “straight/philosophy/wellness” or “mixer/evidence/science” have developed [10, 32, 33]. In response, some suggest that the profession is at a critical crossroad [34, 35]. Others have gone further suggesting the consideration of a complete divorce of the wellness groups from the profession [1, 36].

The notion of referring to “straight” and “mixer” factions of chiropractors in a binary manner is not new [37]. It is even recognised by new chiropractic students [38]. It appears to this observer that the “straight” versus “mixer” categorisation maybe morphing into a more modern binary characterisation of the profession as “wellness” versus “science”, as these new descriptors are used to describe the professional factions. This characterisation is an oversimplification of the educational/scope of practice issues that are better represented as a spectrum of views from strongly wellness/subluxation/vitalist based to strongly evidence/science based [6, 39]. Discussion of the subluxation whilst associated with “wellness” is more a discussion of nomenclature and is beyond the scope of this discussion on the education and scope fo practice of chiropractors. I refer the readers to the following papers for a discussion on Subluxation [40–43].

It is likely that term “wellness” when used to represent chiropractic practice based on straight/philosophy/wellness values might oversimplify a spectrum of views from non-evidence based to evidence-based wellness. Whilst some attempt to represent an evidence-based view of wellness has occurred [6, 44], further supportive evidence is required before wellness-based chiropractic is likely to be accepted by the mainstream within and outside of the profession [43]. To quote Carl Sagan; extraordinary claims require extraordinary evidence.

Despite wellness being a fundamental principle of chiropractic [45] and wellness-related goals being important to many patients and practitioners, they are not important to all [35, 46] as wellness appear to be sought after by a small percentage of the total number of patients presenting to chiropractors [47]. Most patients present for mainly musculoskeletal care [8]. As such, framing a debate about the profession in terms of its philosophy or science belies the fact that a spectrum of beliefs exist rather than two “science” or “philosophy” groups.

Wellness-based practitioners are said to be largely non-evidence based and spine focussed in their management approach [48], which has been confirmed by a recent review which suggested that more effort has been placed into obtaining wellness (primary prevention) than improving musculoskeletal disorders [49]. By contrast, most evidence-based chiropractors focus on musculoskeletal conditions [36, 49] and most of the patients presenting to them are also focussed on spine based MSK conditions [50].

As a reminder, let us note that there are three types of prevention usually described in public health strategies and these include: primary prevention where there is an attempt to prevent getting a disease in the first place, secondary prevention where there is an attempt to recognise a complaint early and prevent it from worsening and tertiary prevention by reducing the symptoms of a disease already present [51]. Claims for the use of wellness-based chiropractic for health require evidence. At present there is little evidence for primary prevention in MSK conditions [52] and particularly in MSK low back pain [53], so it may be challenging for wellness-based chiropractors to create evidence for primary prevention.

It is possible that practitioners that employ a range of primary, secondary and tertiary approaches to prevent spine-based complaints traditionally employed by wellness-based chiropractors may produce evidence if appropriate surveillance systems are used to document outcomes [4]. Clearly, claims currently outstrip evidence in this area and it remains a key goal for this group to validate their claims. A recent best practice guideline in chiropractic health promotion is recommended as a possible way forward [52]. The challenge to the wellness group will be to find evidence to support the teaching of any prevention strategies in evidence-based wellness practitioner training programs. It is key that the pursuit of evidence by the wellness group should be a high priority and that this may be achieved with a partnership with the science /academic group.

As science emerges it is a fundamental requirement of all healthcare practitioners that the new evidence be incorporated into practice, whatever that scope of practice [54]. Science helps to develop a philosophy of practice [55] or should, just as much as the conditions to which it is applied. Hence, scientific method and philosophy are interwoven concepts that continually evolve as their component parts change [56].

This evolution of care is not easily demonstrated within chiropractic particularly as the wellness group deliver care that is a practitioner centric view within a vitalistic construct [32]. By contrast, the evidence-based MSK practitioners have evolved their mechanistic approach to a more multimodal approach leaving behind the strong emphasis on often spine-only adjustment-only management approaches [40] with potential theoretical non-MSK health benefits [56–58]. Ensconced within these factional definitions are variations in the application of traditional chiropractic principles, values and identity that have driven the two tribes approach within the profession [59].

Arguably, some of what is being debated within the chiropractic profession diverges from a patient-centric point-of-view (despite claims to the contrary) [56–58]. As much of the discussion relates back to the professions’ own identity, it seems to be a practitioner driven issue.

### Education by practice management

Modern education in the “wellness” area [60] has often fallen to the technique and practice management groups in lieu of post graduate offerings by independent colleges or universities. Additionally, the term “subluxation” has become associated with wellness and according to some practice management groups, clinical success will be limited without its use [61]. This contrasts to the viewpoint of the “science” group who suggest that subluxation has little or no evidence for it, thus further dividing the groups [62–64]. It is perhaps with such “education” that the association between subluxation, non-symptomatic care, wellness and clinical success derives.

In a near vacuum of tertiary post professional diploma/degree educational offerings in “wellness” by chiropractic institutions have developed tethered programs between some association and entrepreneurial groups of the profession [65]. The entrepreneurial groups continue to promote more traditional views whilst embedding them in various practice management and technique specific goals; some of which are not particularly evidence based [66]. As such, it seems that some of these groups are complicit in the disconnect between entrepreneurial goals without constraint of regulation within traditional tertiary educational settings, whilst other groups appear to be embracing the evidence-based push [67].

Perhaps a key reason for the entrepreneurial delivery of these chiropractic principles lies in the lack of business education within the chiropractic programs [68]. Chiropractic practice balances two key roles: medical/chiropractic ethics and running a small business [69]. Sometimes these goals are not compatible [70] and protocols should be taught to bridge these gaps, ethically [71].

The promotion of traditional (early twentieth century) chiropractic values as a vehicle for entrepreneurial activity in the twenty-first century appears rife within the profession [33]. These values appear to be considered a strength by the traditional, philosophical-based chiropractors within the profession [4] and a weakness by both the MSK end of the profession [33] and medicine [72, 73].

The adoption of this entrepreneurial delivery of education and care has resulted in the castigation of the wellness clinicians by the science clinicians/academics, for not providing appropriate modern, evidence-based practice [1, 35]. However, it could also be argued that the development of this educational component of the entrepreneurial groups grew out of the void created by the absence of valid and representative tertiary educational programs developed to teach wellness from a science-based perspective.

The breakdown of harmony within the profession has resulted from individual groups pushing their own perceived values. The growth/influence (of entrepreneurial groups and lack of externally recognised tertiary education) and divergence of the factions, now underpins calls for the profession to split into two distinct groups [1], reflecting the emergence of a two tribes mentality. This destructive evolution is demonstrated, at least in part, by the recent resignation of senior research personnel from the world governing body (World Federation of Chiropractic or WFC) for reasons that are allegedly related to faction fighting behind-the-scenes and feelings that one group does not represent the other [74].

Perhaps leading university programs could partner with some of the entrepreneur groups in order to foster the development of core competencies of LM to underpin new offerings by the universities [75–77]. Commercial intellectual property arrangements could be one way of hastening the development of programs whilst providing the rigour required by modern healthcare education.

### When two tribes go to war

The wellness group of the profession have largely been driven by entrepreneurial and technique groups that self-represent as the “clinical” end of the profession, whilst the researcher/academic group have largely represented the other group [26, 29].

These two groups, “academic/researcher” and “clinicians” have had long standing differences within and outside of the profession as clinicians and academics frequently do not see eye to eye, resulting in the axiom “Those who can, do; those who can’t, teach” [78, 79].

This division between academics and clinicians seems to accentuate the debate between the entrepreneurial/wellness/philosophy and the academic/evidence/science groups. The controversial role of industry influence in the delivery of medical care and research is not new [80]. Chiropractors, especially those from the “wellness” camp, often talk of this conflict and are quick to denigrate medicine for its links to the pharmaceutical industry yet the chiropractic profession appears to have something of a blind spot as the same could be said to be occurring within its ranks.

At its base, there appears a lack of respect for each group within chiropractic and an inability to unite through respectful open communication of each position using a common governing structure. We must, as a profession, come together in a consensus process to develop a modern method to recognise and promote the differences within chiropractic. A difference that is cognizant of original chiropractic values that are set within modern scientific standards. Standards that serve the different patient populations that chiropractic clinicians serve.

Once achieved, these consensus base standards need to be reflected in messaging within and outside of the profession.

### Don’t mention the war

Those that hold more traditional chiropractic views feel that the modernists are losing their “chiropractic-ness” in the application of the evidence [72]. However, if all practitioners (from different professions) are to apply the science to their respective patient bases, this would surely result in scope of practice approaches that become more similar in time rather than different? I believe we have seen this occur already within the chiropractic profession, as many practitioners are increasingly becoming multimodal in their approach, rather than adopting the more traditional, unimodal perspective (adjustment only) [81–86].

**Fig. 1:**
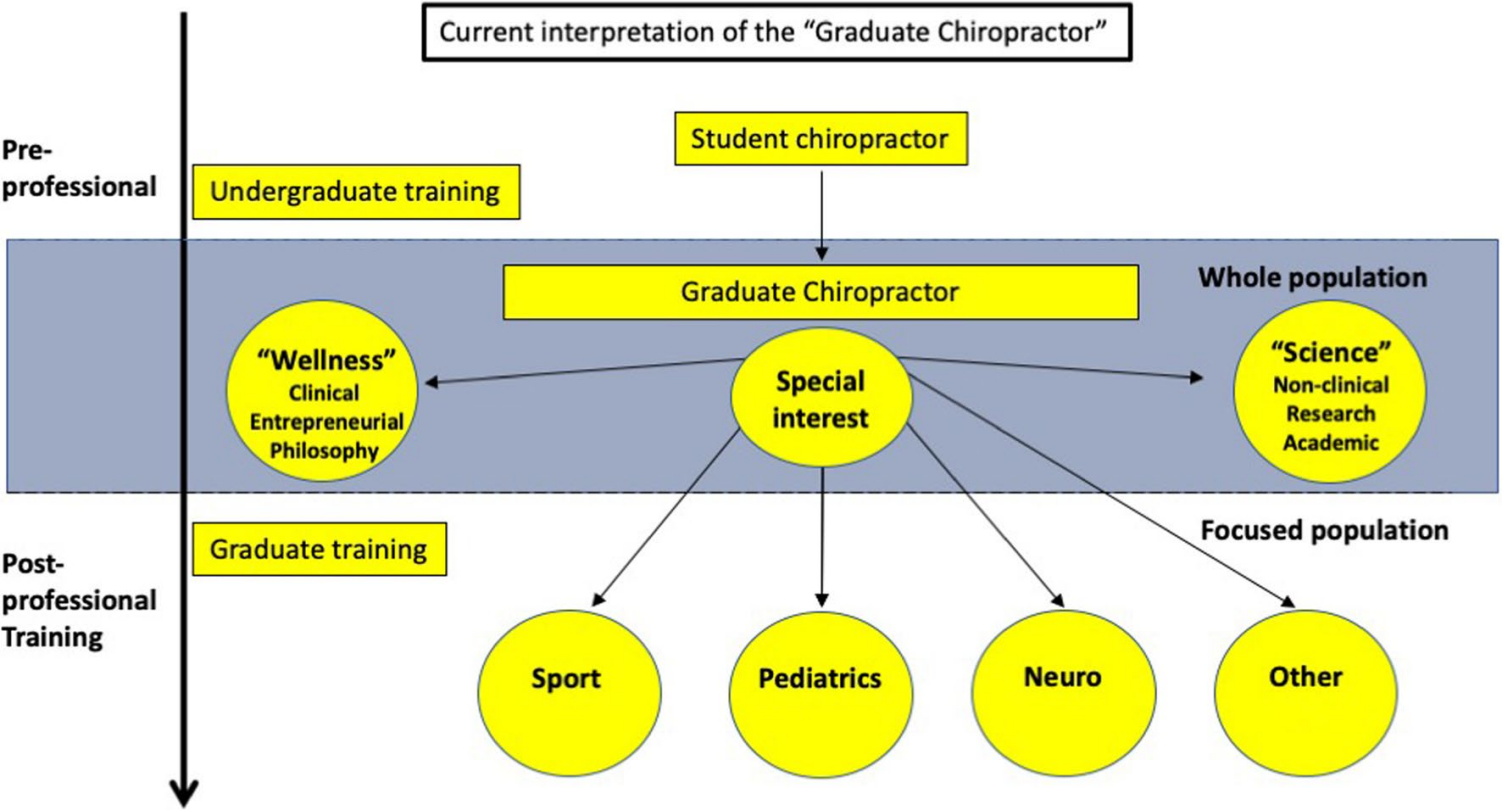
The current view of general chiropractic

A major problem of the difference between the two groups is the concept that graduating chiropractors are somehow different by their orientation (wellness or science) and are somehow differently aligned based on what they believe rather than what they are taught, their competency and who they see as clinicians (See Fig. 1). An issue that has plagued the profession for some time [87].

The profession is large enough and mature enough to house a broad scope of practice, a scope that is inclusive of the many different special interest “flavours” of chiropractic. However, to foster a seamless execution, the scope must be defined by specific competencies in education that are achieved through consensus and must include exposure to appropriate clinical settings, as well as research in relevant management approaches [88]. Having attained the appropriate educational background, these groups will create the extended scope of practice not normally associated with general chiropractic practice, but coveted by those within each of the groups [41].

The different approaches to care are driven by the populations that they serve. Thus, one would reasonably expect the management of a child to be different to an athlete, to an elderly person, or someone without pain who is looking to maximise their health potential. All of these patient subgroups require an evidence informed different “flavour” of chiropractic management to address the specific nature of their presenting clinical problems. By embracing these groups in a consistent manner, one accepts the likelihood that chiropractic management may be different in different groups and that is not only reasonable but required. Therefore, the debate over whether a practitioner is a “pain” practitioner or a wellness (non-pain) practitioner becomes irrelevant. As it should be. The only relevance is the practitioner addressing patient need.

A solution to this ongoing and damaging schism is available to the profession. I propose that the profession introduce an educationally driven competency-based consensus driven standard to *describe* the professional general and special interest groups in both its pre and post profession training in all the populations that it serves. A standard that will describe the level of the training (graduate certificate, graduate diploma, master degree) and is education based not entrepreneurial.

### The structure of the profession

It is flawed reasoning to consider a practitioner as being either a philosophically driven or evidence-based driven. Modern practitioners require both in the management of all patients. To briefly summarise, it is likely in my opinion that the existing schism within the chiropractic profession has developed as a result of many intertwining identity driven issues [10, 34, 89]. These include, but are not limited to:

1. Lack of professional structure to evolve management of sub populations of patients.
2. Lack of “wellness” based education in independent tertiary settings.
3. Initial overly enthusiastic evidence-based medicine push.
4. Pushback by pseudoscience dressed up as philosophy.
5. Too strong a focus on entrepreneurial driven education.
6. Wellness camp presented as “clinicians” and Science camp being represented by “academics”.
7. Lack of documented competencies for post professional special interest education.
8. Promotion of the above being delivered in a way that isolates and divides.

The profession could take a lead from medical and allied health professional educational structures in order to address these issues.

### A new structural model for the profession

A solution for this tiresome and destructive debate is within reach; In times of trouble, “Build bridges, not walls” (Attributed to Suzy Kasem).

More specifically, it is proposed that the way chiropractors describe chiropractic practitioners should be the same as other healthcare professionals. Chiropractors and the chiropractic profession should adopt structural descriptions of practitioners and their populations that are used in other healthcare professions [24, 89–91].

These descriptions should encompass definitions of:

1. Competency
2. Practitioner type
3. Patient population
4. Education

The concept of a general practitioner and a specialist practitioner has been utilised in medicine for a long time. It is also used in other allied health professions. However, in some places like Australia, the term “specialist” is legally considered a term associated with medicine and is not allowed to be used by a chiropractor with or without post graduate training. There the term “special interest” is used in its stead [92].

### Recognising competency

**Fig. 2:**
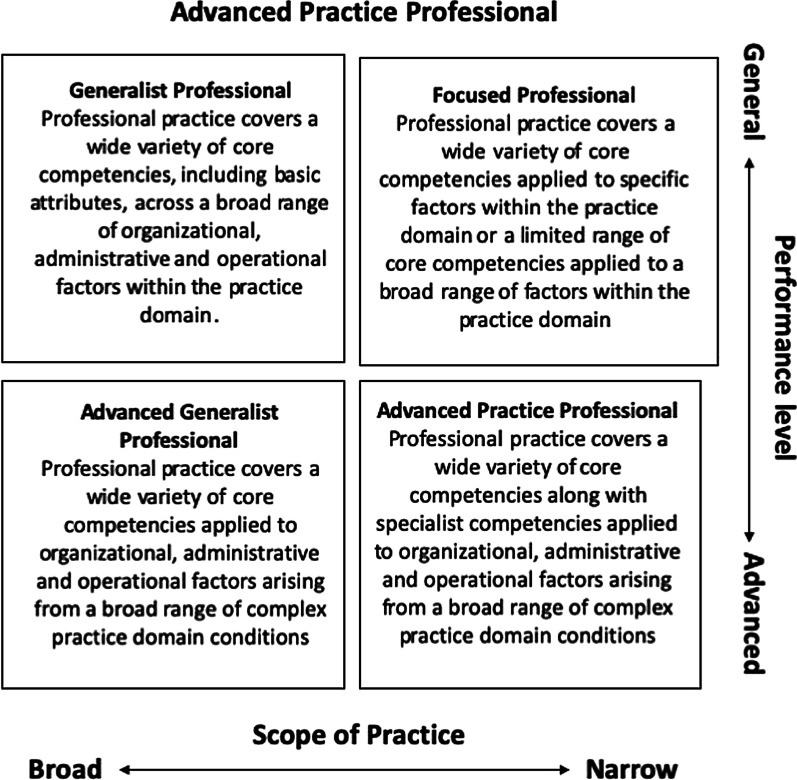
Advanced practice professional [93]

The chiropractic profession is essentially made up of student chiropractors, graduate chiropractors, regulators, associations, insurers, entrepreneurs and others. Upon graduation, chiropractors become general chiropractic practitioners who are typically able to service the entire population. With additional experience and education, new graduates attain greater competency on their way to ultimately becoming highly competent or even expert. See Fig. 2 [93, 94]. An educational standards-based approach is proposed in order to achieve protected title for the various special interest groups in chiropractic.

### Generalists versus specialists

Special interest groups have evolved in chiropractic to meet the need of the profession and the patients that present to it [95, 96]. This has occurred in many locations including the United States [97], Europe [98] and Australasia [99]. Part of this evolution is describing and demonstrating competency, safety and expertise through clinical experience, education, mentoring and research. Establishing evidence for regulators, third party payors, medicine, allied health and patients is key in developing recognised competence and scope, to broaden the patient base presenting to chiropractors in an evidence-based world.

**Fig. 3:**
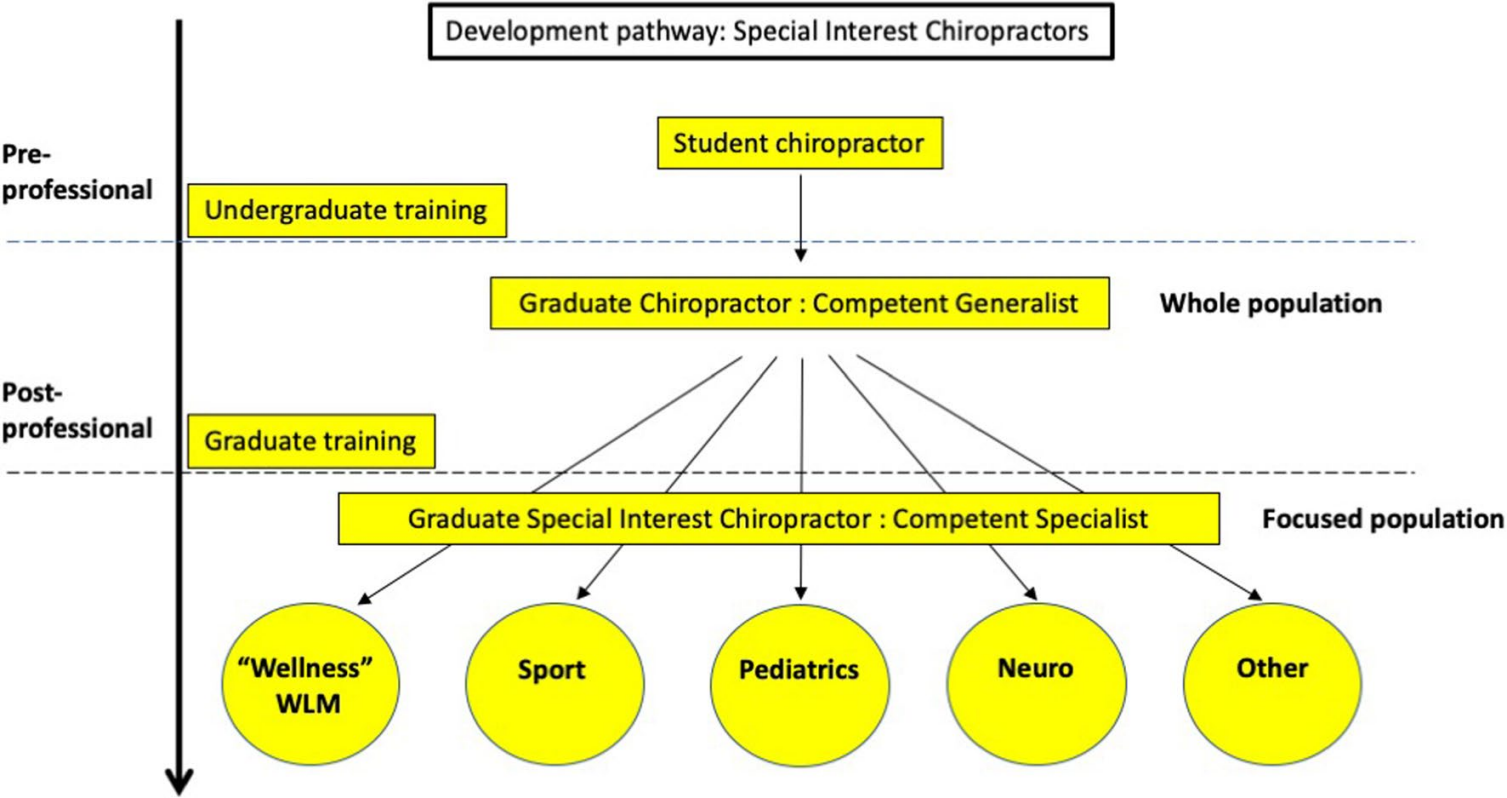
Generalists and specialists

The educational structure required for transition and progression of competence from “competent generalist” to “expert generalist” or “competent specialist” to “expert specialist” is important (see Fig. 3) [100, 101]. Other professions have described themselves in such terms [102–109] and chiropractic has done it with its pre-professional education and some of its post professional specialist training such as Paediatrics [110] with work progressing in the arena of sport and rehabilitation [111]. Structurally describing pre and post professional training in terms of competency would be beneficial in resolving the divide within the “wellness” and “science” camps of the profession by providing definition of scope. Ideally, it would be the role of the professional stakeholder groups via consensus to determine the competencies required for each specialty. Thus, there is a need to describe international best practice in the specialist areas in chiropractic including “wellness”. A model for this training exists in other allied health professions [112, 113].

### Undergraduate chiropractic training

Students are trained to be chiropractors. This training can be referred to as pre-professional education as the term post-graduate can be confusing in jurisdictions where two diplomas or degrees are required for registration. Upon graduation from their undergraduate/pre professional studies, graduates in most jurisdictions enter a profession to be generalists [114, 115]. General chiropractors are, by their training, legally able to treat patients from the entire lifespan [106, 107]. That is: young, middle aged, and old persons, as well as all other subsets of the population including: sports, neurorehabilitation, paediatrics, pain, geriatrics occupational health and safety amongst other special interest groups. This includes wellness focussed patients.

Those practitioners that choose to focus on a particular patient population, for example wellness, do so by choice and are voluntarily restricting or in many cases focussing on a sub population of the whole population of patients.

I propose that wellness populations be considered a chiropractic special interest group and are thus managed in the same manner as other special interest groups.

### The competent general chiropractor

The student chiropractor graduates to become a general chiropractor who is described as a competent general practitioner [116, 117]. This chiropractor can manage patients of the entire lifespan without focus. This type of chiropractor is typically referred to by chiropractors as a primary contact practitioner [118], a view which is questioned by some [119]. A more broadly acceptable definition is of a MSK practitioner that is a limited care practitioner [94, 120].

### The competent specialist chiropractor

The general chiropractor may choose to focus their interest to a particular population such as: sport, paediatrics, neurology or other special interest population. This typically occurs after additional post professional (post graduate chiropractor) training at a college/university [118, 121]. Upon completion of post graduate study (which may require other factors such as experience, mentoring and research) the general chiropractor may graduate to become a competent specialist chiropractor [94].

### The expert specialist chiropractor

The specialist chiropractor can continue to evolve their expertise in areas of education, clinical experience, mentoring and research to acquire “expert specialist” status [122]. There is common acceptance of the term specialist in chiropractic but there is not too much published on the depth and scope of the competencies required to achieve it [123]. Such a status may be reserved for those that have completed post professional masters level education or above in the speciality area plus additional years of experience in the population they serve plus additional mentoring and research including likely extensive theoretical and practical examination as well as clinical experience and research literacy [94, 101].

### The expert general chiropractor

In Australia, the general practice of medicine has become a post professional specialty that elevates the standards of private practice to a specialist status whilst providing increased remuneration from government and other funding sources [124]. Could such a concept work in chiropractic? As with the expert specialist chiropractor, the expert generalist chiropractor (a musculoskeletal specialist with a spine focus) would continue to evolve their expertise in areas of education, clinical experience, mentoring and research to acquire “expert” status. This status would also require post professional education (likely at a master degree level) as the “expert specialist” chiropractor does. There are at least three possible names for this practitioner and all already exist: Musculoskeletal Chiropractor, Orthopaedic Chiropractor or Primary Spine Practitioner depending on the focus of the special interest [89]. Perhaps it is time to focus the interest of practitioners with a strong spine focus by amalgamating some of the practitioner training programs into a spine focussed practitioner and have others move into other special interest areas.

### Vertical integration

Many of the traditional values of the wellness chiropractor are encompassed within modern healthcare paradigms of LM [13, 118].

LM has less to do with direct musculoskeletal intervention and more to do with the application of prevention strategies and the application of public health principles [15, 125]. Should a broad-based scope of practice claimed by some chiropractors [48], be supplemented within WLM practice, the combined approach, when supported by evidence, may serve to modernise the wellness-based practice of chiropractors potentially attracting a broader clientele to the profession. As development in the medical sub-discipline of LM is so recent [75], a musculoskeletal sub-discipline of WLM practice does not yet exist to serve a population of patients potentially awaiting chiropractic involvement. Thus, there is potential to modernise the “wellness” practice of some chiropractors whilst establishing a lead in an evolving area of healthcare.

Evolution in the education of wellness practitioners is occurring in the form of LM [9] and Health Education Specialist [126, 127]. Both of these groups are occurring primarily outside of the chiropractic profession and they serve to illustrate a possible pathway for elements of these sub disciplines to be integrated into a modern wellness-based chiropractic curriculum.

Hopefully the nomenclature of this group is not the next topic of division. Science and terminology evolve as our definitions do. Presenting knowledge in an historical context demonstrates evolution and advancement and this should be a goal of all sections of the profession.

For the sake of accuracy, legitimacy and inclusiveness, I propose that the wellness chiropractor be renamed as a Wellbeing and Lifestyle Medicine clinical chiropractic practice group or something similar. The WLM name is chosen on the basis that it will vertically integrate into a medical sub-specialty just as other special interest groups do. However, the term Wellbeing and Lifestyle Chiropractor (WLC) or Wellbeing and Lifestyle Management (WLM) might be just as fitting as the use of the term”medicine” when referring to chiropractic activity is likely illegal in some jurisdictions as “medicine” is a protected title [128].

Importantly the standards must be driven by minimum international post professional educational standards rather than those standards set by individual private entrepreneurial groups with vested interest. By adopting a consensus approach to the education and recognition of wellness care within the WLM group, this group would compare favourably to any other recognised subgroup within the profession with all the same rights and privileges. It is hoped that such a definitional and operational change will enhance the respect of the WLM group by those that see themselves as championing evidence-based practice.

### Broadening the scope of chiropractic

Recognising the wellness (WLM) group as a specialty within chiropractic will likely ignite a discussion of definition and scope.

By defining and recognising the scopes of different specialist groups within the profession, a broader recognition of the different management approaches used within the groups will be identified. Such intra professional harmony would likely reduce the debate about what, and what is not, chiropractic [64]. Additionally, these groups have the potential to broaden the recognised scope of practice of chiropractic should the extended scope be supported by evidence. This difference should go some way to explaining, and hopefully recognising, that not all chiropractors operate in the same fashion, nor should they.

A system that recognises the general chiropractor, as well as different specialist chiropractors, with minimum educational standards outlined for each would ultimately solidify an extended scope of practice for the chiropractic profession. This should broaden its appeal to the public, and thus, facilitate the utilisation of chiropractic services by the population in general.

### Achieving consensus on standards

The discussion of definitions and standards of care presented in this opinion are a first and important step to develop a “middle ground”. However, this model will need to be developed through a transparent consensus process to begin to operationalize its thinking. An example of this process might include the development of a consensus as represented in the Proceedings of the Mercy Centre Consensus Conference [129]

An early part of this process would involve all parties being invited to participate and be equally represented within that process. It is likely that the adopted level of education to represent post professional specialisation may be different in different jurisdictions due to local competitive, legal and other professional issues. An example of this would be the United States where multiple jurisdictional scenarios exist. However, minimum standards should apply. A consensus could be developed on minimum standards. Whether standards are set at a graduate diploma/diplomate or master degree level [97] will be based on local factors. Additionally, time for the professional jurisdiction to transition will also be important. Establishing a lower initial level that raises to a higher level after the passage of time and the development of supporting infrastructure may be required [98] allowing all concerned an easier path to higher standards.

## Conclusion

The chiropractic profession needs to describe its pre- and post-professional training, its sub-populations of special interest areas, and its scope in terms of professional generalist and specialist competency. It has described its competencies at the pre-professional level, but has not completed the journey in describing competencies for all post professional education special interest areas. In order to address this limitation, the addition of the Wellbeing and Lifestyle Chiropractic/Management clinical practice group to incorporate the traditional views of the wellness-based chiropractor is recommended.

In doing so, the WLM group may be embraced as a wanted and needed member of the profession by the science group as based on modern science. I believe the proposed actions address the divide that has grown in the profession with a workable solution. This solution may reduce the infighting and provide a respectable discourse within a consensus process for all stakeholder groups including the associations, the regulators, the educators, the clinicians and the entrepreneurs.

It seems that many of the actions/values associated with a contemporary patient focussed healthcare profession are potentially contained within this brief. However, the consideration of a “divorce” is extreme and ultimately unnecessary. Instead, with changes in the way chiropractors define themselves, the professional factions may be able to coexist and broaden the recognised scope of chiropractic practice and its appeal to the public and other stakeholders.

“United we stand, divided we fall”.

## Data Availability

Not applicable.
