# Correction to: Reframing a debate in chiropractic

**DOI:** 10.1186/s12998-021-00402-4

**Published:** 2021-11-22

**Authors:** Henry Pollard

**Affiliations:** Department of Chiropractic, School of Medical and Applied Sciences, CQUniversity, Brisbane, Australia

## Correction to: Pollard Chiropr Man Therap (2021) 29:44 10.1186/s12998-021-00401-5

Following the publication of the original article [1], we were notified that two sentences contained information that did not correctly reflect recent events, and therefore have been removed. Reference 73 has been also removed and subsequent references have been renumbered.

The original article has been corrected.
